# Protein Sorting in Plasmodium Falciparum

**DOI:** 10.3390/life11090937

**Published:** 2021-09-09

**Authors:** D.C. Ghislaine Mayer

**Affiliations:** Department of Biology, Manhattan College, Riverdale, New York, NY 10471, USA; ghislaine.mayer@manhattan.edu

**Keywords:** protein sorting/trafficking, vesicular trafficking, endocytic compartment, malaria, apicomplexans

## Abstract

*Plasmodium falciparum* is a unicellular eukaryote with a very polarized secretory system composed of micronemes rhoptries and dense granules that are required for host cell invasion. *P. falciparum*, like its relative *T. gondii*, uses the endolysosomal system to produce the secretory organelles and to ingest host cell proteins. The parasite also has an apicoplast, a secondary endosymbiotic organelle, which depends on vesicular trafficking for appropriate incorporation of nuclear-encoded proteins into the apicoplast. Recently, the central molecules responsible for sorting and trafficking in *P. falciparum* and *T. gondii* have been characterized. From these studies, it is now evident that *P. falciparum* has repurposed the molecules of the endosomal system to the secretory pathway. Additionally, the sorting and vesicular trafficking mechanism seem to be conserved among apicomplexans. This review described the most recent findings on the molecular mechanisms of protein sorting and vesicular trafficking in *P. falciparum* and revealed that *P. falciparum* has an amazing secretory machinery that has been cleverly modified to its intracellular lifestyle.

## 1. Introduction

*Plasmodium falciparum* is a protozoan parasite that is responsible for millions of infections resulting in malaria, a devastating disease currently prevalent in sub-Saharan Africa. Malaria continues to be endemic in 87 countries with approximately 229 million cases and 2–3 million deaths [1]. *Plasmodium falciparum* is an obligate intracellular microbe that infects human erythrocytes. Obligate intracellular microbes use diverse mechanisms to hijack their host cellular processes. Following uptake, most intracellular microbes take over the host organelles to create distinctive and unique microenvironments. Unlike other intracellular microbes, *P. falciparum* has the unique challenge of residing and developing within a cell devoid of organelles.

Inside the erythrocyte, while the parasite resides within the parasitophorous vacuole, it orchestrates the drastic modification of the erythrocyte. After erythrocyte invasion, the composition of the erythrocyte membrane as well as its permeability and rigidity are radically altered [2]. All the symptoms associated with malaria are due to the invasion, intracellular development, and egress of *P. falciparum* merozoites into the host’s erythrocytes [3]. Once inside the erythrocyte, the parasite undergoes asexual reproduction producing 8–26 daughter merozoites that are released to invade fresh erythrocytes [4]. The life cycle of the parasite requires the trafficking of a large number of proteins to the erythrocyte, which are necessary for the complete takeover of the erythrocyte. To enable interactions with the erythrocyte, *P. falciparum* transports virulence proteins using *de novo* specialized secretion systems. The trafficking of virulence factors and their insertions into the erythrocyte membrane at the knob structures are key events in the development of the pathologies associated with severe malaria, both cerebral and placental [5]. Therefore, the pathogenesis *P. falciparum* is dependent on the mechanism of protein sorting and trafficking during the intraerythrocytic stage.

Protein sorting is the process by which proteins are moved to their proper destinations inside and outside the cell [6]. In eukaryotes, proteins are directed to the lumen of specific organelles, diverse intracellular membranes, the plasma membrane, or outside of the cell by the process of secretion [6]. The protein targeting process is controlled by specific sequences within the protein itself [7,8]. In order to successfully replicate inside the erythrocyte, *P. falciparum* needs to correctly sort and traffic its newly-made proteins to all of its organelles. In addition to trafficking proteins within its cell, the parasite ships proteins beyond its plasma membrane, into both the parasitophorous vacuole and into the host erythrocyte cytoplasm and plasma membrane [9,10]. Moreover, the parasite possesses a food vacuole, an acidic organelle where the host cell hemoglobin is digested to amino acids. The food vacuole is also the site of action of the antimalarials chloroquine and quinoline [11]. In *P. faclciparum*, additional organelles named mononemes and exonemes have also been described [12]. The latter seems to play a role in the parasite egress [12].

## 2. Protein Sorting to ER and Golgi

Akin to all eukaryotes, *P. falciparum* proteins that are trafficked to the various organelles contain a canonical signal peptide, a short sequence of hydrophobic amino acids located at the amino terminus. The signal peptide indicates the co-translational transport of these proteins into the endoplasmic reticulum (ER). In *P. falciparum*, the ER is located near the nucleus and appears as ‘‘horn-like’’ projections [8]. Except for the apicoplast-bound proteins, the parasite organellar proteins are transported to the Golgi apparatus [13]. In most eukaryotes, the Golgi apparatus is organized into biochemically well-defined compartments called cisternae termed the *cis*-, medial-, and *trans*-Golgi. In mammalian cells, these cisternae are stacked or in close apposition to each other. However, in the budding yeast, *Saccharomyces cerevisiae*, the Golgi cisternae are unstacked [14]. *Plasmodium falciparum* seems to have a simplified, unstacked Golgi apparatus. N-linked glycosylation, a process that is finalized in the *cis*-Golgi, does not occur in *P. falciparum* [15]. Several ultrastructural studies involving serial sectioning and 3-D reconstruction analysis of *P. falciparum*-infected erythrocytes have not determined the presence of stacked Golgi cisternae in the parasite [16,17,18]. In addition, indirect immunofluorescent studies using antibodies against *cis*- and *trans*-Golgi resident proteins of *P. falciparum* also support the absence of stacked cisternae in *P. falciparum* [19,20]. Therefore, in *P. falciparum*, the protein sorting apparatus from the ER to Golgi appears to be reduced.

## 3. Protein Sorting to the Apicoplast

**Figure 1:**
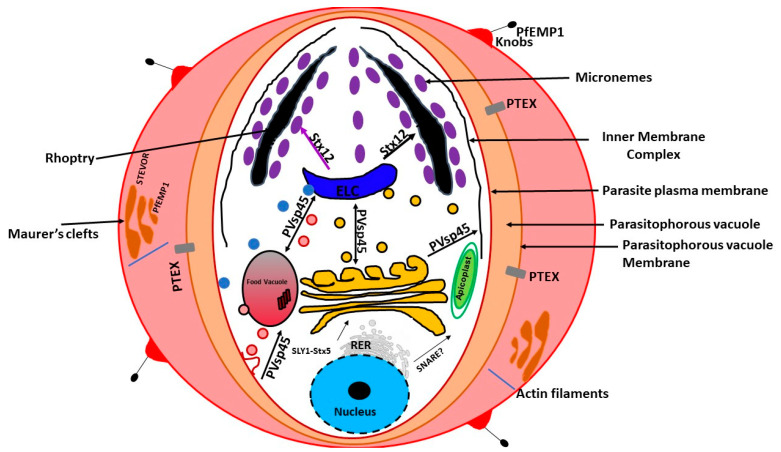
Schematic representation of protein trafficking directed by *P. falciparum* merozoite-infected erythrocyte. The model outlined in this figure is a summary of the recent data on protein sorting and trafficking in *P. falciparum* and its relative, *T. gondii*. Secreted proteins are made in the rough ER (RER). Secretory vesicles from the RER are thought to be shipped to the cis-Golgi with the help of SLY1 and Stx5. Bidirectional sorting between the endo-lysosomal compartment (ELC) and the trans-Golgi network (TGN) is also mediated by Vsp45. Transport from the ER to the apicoplast is facilitated by unidentified SNARE proteins. Proteins destined to the micronemes and rhoptries are sorted by the adaptor Stx12. Endocytosis, which leads to the host cell cytosol uptake (HCCU), is likely to occur via the cytostome or the micropore (not shown). These vesicles are shuttled to ELC. The SNARE proteins involved in this process are unknown. Vsp45 is necessary for endocytosis and release of the cargo to the food vacuole. The knobs and some of the proteins involved in PfEMP1 trafficking are shown.

In the mid-1990s, a unique organelle characterized by the presence of a vestigial, non-photosynthetic plastid was discovered in *P. falciparum* [21,22,23]. The apicoplast in *P. falciparum* seems to be positioned on a small projection of the ER, but it is not clear whether it is part of the ER [13]. However, current evidence suggests that the apicoplast is separate from the *cis*-Golgi [13]. In addition, the apicoplast appears to be located upstream of the *cis-*Golgi (Figure 1). The apicoplast is surrounded by four membranes and is thought to be the result of secondary endosymbiosis [24]. It is the site of quite a few metabolic pathways, such as heme, isoprenoids, and fatty acids syntheses [25]. It is thought that the outermost membrane of the apicoplast is part of the endomembrane system [24]. Most of the resident apicoplast proteins are encoded by genes located in the parasite nucleus [26,27]. These proteins are translocated across the four membranes by means of a two-part N-terminal extension that has been shown to be both necessary and sufficient for sorting to the apicoplast [24]. The first segment of the N-terminal exten sion sequence is similar to a classical signal peptide. It is responsible for the translocation into the secretory pathway. On the other hand, the second segment shares homology to chloroplast transit peptides and is needed for entering the apicoplast [28].

Previous experiments using green fluorescent reporter in which the apicoplast signal peptide protein was replaced by the signal peptide of a micronemal and a knob-associated protein indicated that targeting proteins to the apicoplast begins at the general secretory pathway of *P. falciparum* [13]. In *P. falciparum,* the signal peptide in apicoplast-resident proteins, was shown to facilitate translocation across the rough ER (RER) membrane through the Sec machinery releasing the protein into the ER lumen [29,30]. This is followed by the removal of the signal peptide by signal peptidase [31,32]. Once in the ER, the transit peptide directs the transport into the apicoplast [28]. This mechanism of trafficking seems to be shared by other secondary plastids [31,32,33,34,35]. Furthermore, it has been validated in *Toxoplasma gondii* [36,37,38,39]. On the other hand, apicoplast transmembrane proteins that are encoded by the nucleus are trafficked via the Golgi apparatus [40].

## 4. Protein Sorting to Food Vacuole

The intraerythrocytic stage of *P. falciparum* depends heavily on the host cytosolic proteins, which are funneled to the parasite by endocytosis. Hemoglobin from the erythrocyte cytosol is shuttled across the parasitophorous vacuole membrane (PVM), the plasma membrane of the parasite, via the cytostome or micropore [41,42]. Their final destination is the food vacuole, a lysosomal-like compartment in *P. falciparum* that is specialized for degradation. In *Toxoplasma gondii*, this organelle is called the lysosomal-like vacuolar compartment or VAC [43,44]. During the intraerythrocytic stage, *P. falciparum* takes up a considerable portion of the host cell cytosol. The erythrocyte cytosol containing mostly hemoglobin is carried to the food vacuole where it is digested, providing the necessary amino acids required for growth and development [45,46,47]. There, the ingested hemoglobin is acidified and broken down to amino acids. In the food vacuole, peptides are transported and heme is polymerized and detoxified. The food vacuole is also the site of action of antimalarials such as chloroquine. The host cell cytosol uptake (HCCU) that leads to the formation of the food vacuole is essential for the parasite’s survival.

Although many of the resident food vacuole proteins have been well-characterized, the molecular mechanism of protein sorting to the food vacuole is not well understood [12]. Protein trafficking from the host cytosol to the food vacuole of *P. falciparum* and the vacuole (VAC) of *T. gondii* remain elusive. It was previously thought that the cytostome was the main driver for the formation of the HCCU [48]. Experiments using inhibitors of the cytoskeletal proteins actin and myosin, as well as inhibitors of *soluble N*-*ethylmaleimide*-*sensitive factor attachment* protein *receptor* (SNARE) and dynamin, had suggested that they could play a critical role in the formation of HCCU [49,50,51,52]. However, data from a recent study did not seem to implicate these cytoskeletal proteins. Instead, the *P. falciparum* vacuolar protein sorting-associated protein 45, PfVPS45, was shown to be required for the formation of HCCU [53]. Conditional mutants of the *pfvpd45* gene resulted in the buildup of vesicles packed with the host cell cytoplasm [53]. These vesicles seem to be connected to the host cell [53]. VPS45 was further shown to be needed for trafficking from the host cell cytosol to the food vacuole [53]. In addition, hemoglobin transport to the food vacuole was inhibited in the mutants, halting parasite growth [53]. This function is conserved in *T. gondii* where TgVPS45 mediate VAC digestion of host-endocytosed proteins [54].

Interestingly, many of the HCCU vesicles seem to have endosomal features since they contain phosphatidylinositol 3-phosphate [53]. In other organisms, such as the budding yeast, VPS45 plays a role in endo-lysosomal transport [55,56]. It appears that *P. falciparum* is similar to its apicomplexan counterpart, *T. gondii*, in using the machinery of the endo-lysosomal system for secreting protein [57,58,59,60,61,62,63,64,65,66]. Rab5 plays a central role in early endocytosis where it moves its target to the early endosomes, transports it to the endosomes, and mediates internalization of receptors. However, *T. gondii* Rab5 paralogs have not been shown to be involved in endocytosis [61]. Rather, they are involved in protein secretion [61]. In summary, the endosome-like compartment (ELC) is where endocytosis and exocytosis overlap in apicomplexans [62]. Protein sorting at the ELC is coordinated by several tethering proteins, such as clathrin [67], HOPS, CORVET [68], DrpB [57], and the Rab5 paralogs in *T. gondii* [54,61].

## 5. *P. falciparum* Protein Export

Following infection, *P. falciparum* releases the contents of its apical organelles, leading to the formation of the parasitophorous vacuolar membrane (PVM), within which it inhabits. Although the PVM provides protection to the parasite, it is also an obstacle for the transfer and distribution of the host-targeted effectors. These effector proteins can either be shipped to or across the parasite-plasma membrane (PM), as well as the lumen of the PVM. These effectors comprise *P. falciparum* erythrocyte membrane protein 1 (PfEMP1) that is secreted to the erythrocyte membrane. It is estimated that *P. falciparum* secretes over 400 proteins into the erythrocyte [69]. These proteins alter the structure and physiology of the erythrocyte membrane, leading to its rigidity and changing its permeability [70]. The proteins that the parasite ships to the erythrocyte membrane are needed for nutrient uptake, evasion of the host immune, and for release from the infected erythrocytes [71,72,73].

During the erythrocytic cycle, *P. falciparum* causes significant and drastic changes to the erythrocyte cytoplasm and plasma membrane, leading to the formation of the Maurer’s clefts and protrusions in the membrane called knobs. Maurer’s clefts are disc-shaped cisternae that are moveable in the cytoplasm during the ring intraerythrocytic stage but later become attached to the erythrocyte membrane skeleton [74]. The Maurer’s clefts are bound to the erythrocyte membrane by a complex of proteins composed of the host actin filaments and parasite-encoded proteins [75,76]. The knobs function as a scaffold for the display of the virulence antigen *P. falciparum* erythrocyte membrane protein 1 (PfEMP1) on the erythrocyte plasma membrane [76].

PfEMP1 mediates the sequestration of the infected erythrocytes to tissues and to uninfected erythrocytes, leading to anemia and severe symptoms [74]. PfEMP1 is responsible for the attachment of infected erythrocytes to endothelial lining of the blood vessels, causing the infected erythrocytes to sequester away, thus avoiding clearance from the spleen [2]. PfEMP1 is shipped into the erythrocyte cytoplasm through the *Plasmodium* translocon of exported proteins (PTEX) located on the parasitophorous vacuole membrane [77,78]. The present model proposes that PfEMP1 is first trafficked to the Maurer’s clefts in association with the chaperone complex made up of hsp70/hsp40 in a soluble state [79,80]. Recently, two PfEMP1-interacting complexes were described [81]. The *P. falciparum* GEXP07/CX3CL1-binding protein 2 (CBP2) was shown to be necessary for PfEMP1 trafficking to the erythrocyte membrane [81]. Deletion of GEXP07 results in parasite with distorted Maurer’s clefts, faulty PfEMP1 trafficking, abnormal knob formation, and failure of infected erythrocytes to attach to the endothelial lining [81]. The mutant parasites seem to grow faster than the wild-type parasites. Moreover, erythrocytes infected with the mutant parasite are less rigid and more vulnerable to osmotic pressure [81]. It has been hypothesized that vesicle-containing PfEMP1 bud from the Maurer’s clefts and travel along the host actin filaments [82]. However, the precise mechanism of PfEMP1 trafficking remains to be elucidated.

The export of *P. falciparum* proteins into the erythrocyte begins in the parasite ER. A subgroup of parasite-exported proteins contains an N-terminal export signal named *Plasmodium* Export Element (PEXEL), or the Host-Targeting motif [83,84]. The PEXEL motif is a pentameric sequence (RxLxE/Q/D) that is cut during translation at the conserved leucine residue by plasmepsin V, an ER-resident endoprotease [85,86,87]. It was recently demonstrated that early recognition of exported proteins takes place during transit across the ER via interactions of plasmepsin V and molecules of the translocation machinery [88]. This indicates that plasmepsin V plays the role of signal peptidase for PEXEL-positive exported proteins. However, the following steps responsible for export have not been defined. A large number of proteins which lack the PEXEL/HT motif are exported to the erythrocyte [89,90,91]. These so-called PEXEL-negative exported proteins (PNEPs) are thought to use some of the same export machinery as the PEXEL-positive proteins [77,78,89,90]. The mechanism by which parasite proteins are exported and refolded in the host cells remains largely unknown. Current data suggest that parasite-exported chaperones as well as chaperones from the host erythrocyte are involved in the process. Indeed, *P. falciparum*-exported Hsp40 co-chaperones have been shown to play a critical role in remodeling the erythrocyte [92]. Moreover, the localization of erythrocyte Hsp70 was demonstrated to be altered following infection with *P. falciparum,* since it is soluble in uninfected red blood cells, but is associated with detergent-resistant fractions after infection [93].

Exported *P. falciparum* proteins must also go through the PVM. This is accomplished by the *Plasmodium* translocon of exported proteins (PTEX) [94,95]. The translocon is a multi-complex structure composed of EXP2, an integral membrane protein, PTEX150, and the AAA+ ATPase Hsp101, a chaperone [96,97,98]. As an AAA+ ATPase, Hsp101 couples ATP hydrolysis with protein unfolding as the exported protein moves through the translocon [98]. It plays a critical role in the export of parasite proteins [98]. It has been shown that exported proteins that are kept in the folded state remain stuck in the parasite vacuole and block the PTEX translocon [77,95,99,100,101]. These data indicate that PTEX functions as a gateway for *P. falciparum* proteins to the host cell, and that unfolding by chaperones play a crucial role in the export process.

## 6. Vesicular Trafficking in *P. falciparum*

In eukaryotes, communication between organelles is carried out by budding vesicles that move along the cytoskeleton to attach and fuse with their target membranes [102]. The accuracy of the process depends on three molecules that act as distinctive identifiers: the SNARE proteins, the small GTPases, and the phosphatidylinositol phospholipids [103,104]. The assembly of the SNARE complex is regulated by Sec1/Munc18-like proteins in coordination with the syntaxin (Stx)-like SNARE protein [105]. The role of Sec1/Munc18 and SNARE proteins in *P. falciparum* and *T. gondii* was recently examined in order to discover the molecules involved in vesicular trafficking in these two apicomplexan parasites. The genome of apicomplexan parasites encodes for four Sec1/Munc18 proteins, including the orthologue of the suppressor of loss of YPT1 function (SLY1), which was shown to be necessary for the movement of vesicles from the ER to the Golgi [53]. SLY1 controls the secretion of all proteins from the Golgi. In apicomplexans, it was determined that Sec1/Munc18 interacts with SNARE proteins to trigger vesicle targeting to numerous organelles, including the pre-rhoptries, the micronemes, the vacuolar compartment, the apicoplast, as well as the inner membrane complex (IMC), a membrane-cytoskeletal scaffold made of flattened vesicles thought to have originated from the *trans*-Golgi [53,106,107]. Sec1/Munc18-controlled vesicles are thought to originate either from the ER, the Golgi apparatus, or the endosomal-like compartment (ELC) [53]. The ELC is where proteins targeted to the micronemes and the rhoptries are sorted and it is also where micronemal and rhoptry proteins are processed by aspartyl protease 3 (ASP3) [59,108]. It has recently been demonstrated that Vps45 in both *P. falciparum* and *T. gondii* is responsible for the biogenesis of the IMC [53]. It is hypothesized that Stx16 and Stx6 work with Vps45 in the formation of the IMC [53]. Additionally, the sorting of the rhoptry, micronemal, and apicoplast proteins to their respective organelles was shown to be dependent on Stx12, an ELC resident protein [53].

The current model of vesicular trafficking in apicomplexan parasites suggest that secreted proteins are first synthesized in the rough endoplasmic reticulum [54,62]. The proteins are then transported to the *cis*-Golgi, likely by SLY1. The fusion of these vesicles is thought to be mediated by Stx5, a protein known to interact with SLY1 [109]. On the other hand, the inner membrane complex was shown to be derived from the *trans*-Golgi compartment, where Vsp45 in coordination with Stx16 and Stx6 is involved in the re-processing of the trafficking factors used during biogenesis of the inner membrane complex [53]. Likewise, Vsp45, in association with Stx16 and Stx6, is also responsible for trafficking between the ELC and the *trans*-Golgi [110]. Although homeostasis of the apicoplast requires Stx12, the SNARE proteins responsible for transport of apicoplast proteins to the ER have not yet been identified [53]. In summary, the identity of specific proteins responsible for trafficking to the various *P. falciparum* organelles have not fully been uncovered. The current data suggest that endocytosis takes place either in the micropore or the cytostome [41,42,49], followed by transport into sub-compartments of the ELC [62]. Vsp45 and most likely Stx16 are involved in this process [53]. The SNARE proteins involved in the endocytic process have not been identified.

## 7. The Role of Prenylation in *P. falciparum* Vesicular Trafficking

The rhoptries and micronemes of apicomplexan parasites are thought to originate from the post-Golgi vesicles. The adaptor protein (AP) complex has been shown to play an important role in protein trafficking to the rhoptry [111]. This complex is responsible for cargo recognition during post-Golgi trafficking in eukaryotic cells [112]. It is a heterotetrameric complex composed of subunits of varying sizes that have distinct roles in the trafficking process [113,114,115]. They are organized into two large subunits (γ, α, δ, ε, ζ, and β1–5), a medium (μ1–5), and a small subunit (σ1–5). Five adaptor protein complexes have been described [108]. AP-1 and AP-2 are known to recognize clathrin, whereas AP-4 and AP-5 do not; the interaction of AP-3 with clathrin has not been clearly established [116]. In higher eukaryotes, the AP-1 complex mediates trafficking between the *trans-*Golgi network and the endosome, while AP-2 is responsible in clathrin-mediated endocytosis [113]. In contrast, AP-3 directs trafficking to the lysosomes [113]. The roles of the AP-4 and AP-5 complexes remain unclear, although some studies suggest that they might be involved in the shipment of cargo to the endosomes [112,117]. In *P. falciparum*, the AP-1 complex was shown to be necessary in the trafficking of rhoptry proteins [118]. Indeed, the medium subunit of *P. falciparum*, Pfμ1, was shown to colocalize with Golgi/ER markers in the trophozoite stage, but Pfμ1 was later associated with the rhoptry makers, RAP1 and Clag3.1 [111]. Stx12 has been identified as the SNARE protein responsible for trafficking of both microneme and rhoptry proteins to their appropriate destination [62].

## 8. Vesicular Trafficking in *P. falciparum*

In eukaryotic cells, vesicle fusion depends on the attachment of the SNARE receptors to specific organelles [118,119,120]. Although most SNARE proteins have a C-terminal transmembrane domain that mediates their attachment to their target membranes, some lack these transmembrane domains. The latter rely on lipid modification in order to be membrane-anchored. Protein prenylation is the attachment of either a farnesyl (15-carbon) or a geranylgeranyl (20-carbon) isoprenoid group to cysteine residue of CAAX motif-containing proteins [121,122,123,124]. Protein prenylation is carried out by three groups of protein prenyltransferases: farnesyltransferase (FT), geranylgeranyl- transferase 1 (GGT1), and Rab geranylgeranyltransferase (RabGGT) [125]. The *P. falciparum* prenylated proteome has been characterized [126]. Interestingly, several prenylation candidates were uncovered [126]. Specifically, The SNARE protein PfYkt6p was characterized in *P. falciparum* and shown to be both prenylated and surprisingly geranylgeranylated [127]. Importantly, the data from this study indicate that the transport of Ykt6 in *P. falciparum* depends on prenylation. It is also the first evidence of protein geranylgeranyltransferase activity on SNARE proteins [127]. Taken together, prenylation might play an important role in *P. falciparum* vesicular trafficking. More studies are needed to uncover the role of prenylation in the endocytic and secretory pathway of *P. falciparum*.

## 9. Conclusions

As in other eukaryotes, vesicular trafficking in *P. falciparum* is essential for the creation of organelles, as well as for the communication between organelles. The pathogenesis of *P. falciparum* depends heavily on vesicular trafficking processes. This process is mediated by Rab GTPases [54], phospholipids [72,110], and the SNARE proteins [53]. The ELC plays a central role in *Plasmodium* protein sorting and trafficking. It is now evident that the ELC represents the junction of the endocytic and exocytic, controlling the formation of numerous organelles that are part of these two opposite pathways. It accepts and ships vesicles from the Golgi apparatus to the micronemes, the prorhoptries, and the food vacuole (Figure 1). It is also involved in endocytosis of host proteins. It has recently been shown that Vps45, in both *P. falciparum* and *T. gondii*, is responsible for shipping both the endosomal and secretory vesicles. Additionally, it is required in the formation of the inner membrane complex. In summary, *P. falciparum*, like T. gondii, repurposes a portion of the endosomal system to the secretory pathway particularly for the creation of the secretory organelles such as the micronemes and the rhoptries, which are required during the process of erythrocyte invasion. The SNARE and Sec1/Munc18 proteins in apicomplexans are responsible for creating the ZIP code required for vesicular trafficking. Finally, *P. falciparum* vesicular trafficking appears to be very similar to that of T. gondii, suggesting that the trafficking and sorting pathways are conserved in apicomplexans [52]. Although significant progress has been made in elucidating vesicular trafficking in *P. falciparum*, there still remain many unanswered questions. The tethering molecules responsible for sorting to each of the parasite’s respective organelles have yet to be identified.

## Data Availability

Not applicable.
